# (2*E*)-1-(6-Chloro-2-methyl-4-phenyl­quinolin-3-yl)-3-phenyl­prop-2-en-1-one

**DOI:** 10.1107/S160053681002386X

**Published:** 2010-06-26

**Authors:** A. J. Viji, S. Sarveswari, V. Vijayakumar, Kong Wai Tan, Edward R. T. Tiekink

**Affiliations:** a Organic Chemistry Division, School of Advanced Sciences, VIT University, Vellore 632 014, India; b Department of Chemistry, University of Malaya, 50603 Kuala Lumpur, Malaysia

## Abstract

In the title compound, C_25_H_18_ClNO, the conformation about the C=C double bond is *E*. Significant twists are evident in the mol­ecule, with the benzene ring forming a dihedral angle of 53.92 (11)° with the quinolinyl residue. Further, the chalcone residue is approximately perpendicular to the quinolinyl residue [C_q_—C_q_—C_c_—O_c_ torsion angle = −104.5 (3)°, where q = quinolinyl and c = chalcone]. In the crystal, the presence of C—H⋯O and C—H⋯π inter­actions leads to supra­molecular layers lying parallel to (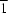
02).

## Related literature

For the biological activity of quinoline derivatives, see: Campbell *et al.* (1998 ▶). For the biological activity of chalcone derivatives, see: Chen *et al.* (2001 ▶); Zi & Simoneau (2005 ▶). For a related structure, see: Prasath *et al.* (2010 ▶).
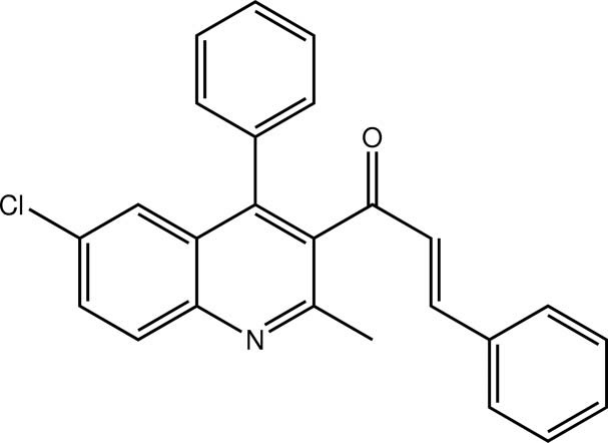


## Experimental

### 

#### Crystal data

C_25_H_18_ClNO
*M* *_r_* = 383.85
Monoclinic, 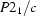

*a* = 9.9250 (9) Å
*b* = 11.1001 (9) Å
*c* = 17.4651 (15) Å
β = 97.250 (1)°
*V* = 1908.7 (3) Å^3^
*Z* = 4
Mo *K*α radiation
μ = 0.22 mm^−1^
*T* = 100 K
0.46 × 0.30 × 0.26 mm

#### Data collection

Bruker SMART APEX CCD diffractometer
Absorption correction: multi-scan (*SADABS*; Sheldrick, 1996 ▶) *T*_min_ = 0.536, *T*_max_ = 1.000
16152 measured reflections
3948 independent reflections
3030 reflections with *I* > 2σ(*I*)
*R*_int_ = 0.079

#### Refinement

*R*[*F* ^2^ > 2σ(*F* ^2^)] = 0.065
*wR*(*F* ^2^) = 0.188
*S* = 1.09
3948 reflections
254 parameters
H-atom parameters constrained
Δρ_max_ = 0.85 e Å^−3^
Δρ_min_ = −0.49 e Å^−3^

### 

Data collection: *APEX2* (Bruker, 2008 ▶); cell refinement: *SAINT* (Bruker, 2008 ▶); data reduction: *SAINT*; program(s) used to solve structure: *SHELXS97* (Sheldrick, 2008 ▶); program(s) used to refine structure: *SHELXL97* (Sheldrick, 2008 ▶); molecular graphics: *ORTEP-3* (Farrugia, 1997 ▶) and *DIAMOND* (Brandenburg, 2006 ▶); software used to prepare material for publication: *publCIF* (Westrip, 2010 ▶).

## Supplementary Material

Crystal structure: contains datablocks global, I. DOI: 10.1107/S160053681002386X/hb5510sup1.cif

Structure factors: contains datablocks I. DOI: 10.1107/S160053681002386X/hb5510Isup2.hkl

Additional supplementary materials:  crystallographic information; 3D view; checkCIF report

## Figures and Tables

**Table 1:** Hydrogen-bond geometry (Å, °) *Cg*1 is the centroid of the N1,C10–C12,C17,C18 ring.

*D*—H⋯*A*	*D*—H	H⋯*A*	*D*⋯*A*	*D*—H⋯*A*
C5—H5⋯O1^i^	0.95	2.48	3.315 (3)	146
C21—H21⋯*Cg*1^ii^	0.95	2.71	3.459 (3)	137

Symmetry codes: (i) 
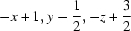
; (ii) 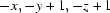.

**Fig. 1.**
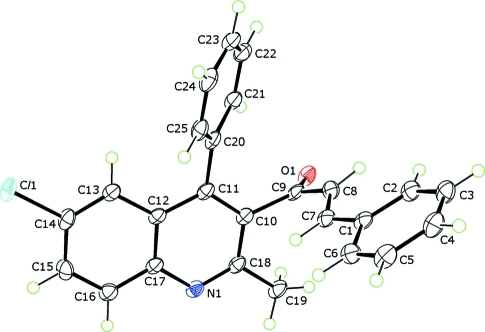
The molecular structure of (I) showing displacement ellipsoids at the 50% probability level.

**Fig. 2.**
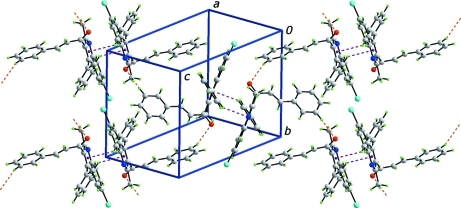
A view of the supramolecular array in (I) highlighting the C–H···O and C–H···π interactions as orange and purple dashed lines, respectively.

## supplementary crystallographic information

### Comment

Both quinolines (Campbell *et al.*, 1998) and open chain flavonoids, *i.e.* chalcones (Chen *et al.*, 2001; Zi & Simoneau, 2005), are known to possess a wide range of biological activities. Herein, in continuation of previous studies (Prasath *et al.*, 2010), we describe the crystal structure of a molecule containing both quinoline and chalcone residues, namely, the title compound, (I).

In (I), Fig. 1, the quinolinyl residue is planar [r.m.s. = 0.041 Å] with both the benzene ring and chalcone residue being twisted out of the plane. The dihedral angle formed between the quinolinyl and benzene (C20–C25) rings is 53.92 (11) °. In the case of the chalcone residue, the twist is best illustrated by the O1–C9–C10–C11 torsion angle of -104.5 (3) °. There are also twists within the chalcone residues as exemplified by the C7–C8–C9–O1 and C7–C8–C9–C10 torsion angles of -163.7 (3) and 14.7 (4) °, respectively. The conformation about the C7═C8 bond [1.340 (4) Å] is E.

Supramolecular layers parallel to (1 0 2) are evident in the crystal structure. These, Fig. 2 and Table 1, are stabilized by C–H···O contacts and C–H···π interactions where the π-system is the NC_5_ ring of the quinolinyl residue.

### Experimental

A mixture of 3-acetyl-6-chloro-2-methyl-4-phenylquinoline (0.01 *M*), benzaldehyde (0.0 1*M*) and a catalytic amount of KOH in distilled ethanol (50 ml) was stirred for about 12 h. The resulting mixture was concentrated to remove ethanol, poured on to ice and neutralized with dilute acetic acid. The solid that formed was filtered, dried, purified by column chromatography using a 1:1 mixture of ethyl acetate and petroleum ether, and recrystallized using ethyl acetate to produce colourless blocks of (I); Yield: 65% and m.pt: 400 K.

### Refinement

The C-bound H atoms were geometrically placed (C–H = 0.95–0.98 Å) and refined as riding with *U_iso_*(H) = 1.2–1.5*U_eq_*(C).

### Crystal data

**Table:**

C_25_H_18_ClNO	*F*(000) = 800
*M**_r_* = 383.85	*D*_x_ = 1.336 Mg m^−^^3^
Monoclinic, *P*2_1_/*c*	Mo *K*α radiation, λ = 0.71073 Å
Hall symbol: -P 2ybc	Cell parameters from 4382 reflections
*a* = 9.9250 (9) Å	θ = 2.2–28.1°
*b* = 11.1001 (9) Å	µ = 0.22 mm^−^^1^
*c* = 17.4651 (15) Å	*T* = 100 K
β = 97.250 (1)°	Block, colourless
*V* = 1908.7 (3) Å^3^	0.46 × 0.30 × 0.26 mm
*Z* = 4	

### Data collection

**Table:**

Bruker SMART APEX CCD diffractometer	3948 independent reflections
Radiation source: fine-focus sealed tube	3030 reflections with *I* > 2σ(*I*)
graphite	*R*_int_ = 0.079
ω scans	θ_max_ = 26.5°, θ_min_ = 2.1°
Absorption correction: multi-scan (*SADABS*; Sheldrick, 1996)	*h* = −12→12
*T*_min_ = 0.536, *T*_max_ = 1.000	*k* = −13→13
16152 measured reflections	*l* = −21→21

### Refinement

**Table:**

Refinement on *F*^2^	Primary atom site location: structure-invariant direct methods
Least-squares matrix: full	Secondary atom site location: difference Fourier map
*R*[*F*^2^ > 2σ(*F*^2^)] = 0.065	Hydrogen site location: inferred from neighbouring sites
*wR*(*F*^2^) = 0.188	H-atom parameters constrained
*S* = 1.09	*w* = 1/[σ^2^(*F*_o_^2^) + (0.0925*P*)^2^ + 1.6015*P*] where *P* = (*F*_o_^2^ + 2*F*_c_^2^)/3
3948 reflections	(Δ/σ)_max_ < 0.001
254 parameters	Δρ_max_ = 0.85 e Å^−^^3^
0 restraints	Δρ_min_ = −0.49 e Å^−^^3^

### Special details

**Table:**

Geometry. All s.u.'s (except the s.u. in the dihedral angle between two l.s. planes) are estimated using the full covariance matrix. The cell s.u.'s are taken into account individually in the estimation of s.u.'s in distances, angles and torsion angles; correlations between s.u.'s in cell parameters are only used when they are defined by crystal symmetry. An approximate (isotropic) treatment of cell s.u.'s is used for estimating s.u.'s involving l.s. planes.
Refinement. Refinement of *F*^2^ against ALL reflections. The weighted *R*-factor *wR* and goodness of fit *S* are based on *F*^2^, conventional *R*-factors *R* are based on *F*, with *F* set to zero for negative *F*^2^. The threshold expression of *F*^2^ > 2σ(*F*^2^) is used only for calculating *R*-factors(gt) *etc*. and is not relevant to the choice of reflections for refinement. *R*-factors based on *F*^2^ are statistically about twice as large as those based on *F*, and *R*- factors based on ALL data will be even larger.

### Fractional atomic coordinates and isotropic or equivalent isotropic displacement parameters (Å^2^)

**Table:**

	*x*	*y*	*z*	*U*_iso_*/*U*_eq_	
Cl1	0.10119 (8)	−0.00729 (6)	0.39598 (4)	0.0310 (2)	
O1	0.13396 (19)	0.68284 (16)	0.58922 (12)	0.0270 (5)	
N1	0.3459 (2)	0.4751 (2)	0.42881 (14)	0.0225 (5)	
C1	0.4920 (3)	0.5159 (2)	0.77131 (17)	0.0218 (6)	
C2	0.4665 (3)	0.5785 (2)	0.83726 (17)	0.0254 (6)	
H2	0.3927	0.6335	0.8343	0.031*	
C3	0.5471 (3)	0.5616 (2)	0.90671 (18)	0.0275 (6)	
H3	0.5278	0.6040	0.9513	0.033*	
C4	0.6565 (3)	0.4825 (2)	0.91160 (18)	0.0284 (7)	
H4	0.7118	0.4706	0.9595	0.034*	
C5	0.6847 (3)	0.4212 (2)	0.84662 (18)	0.0291 (7)	
H5	0.7599	0.3677	0.8497	0.035*	
C6	0.6033 (3)	0.4376 (2)	0.77700 (17)	0.0254 (6)	
H6	0.6233	0.3952	0.7325	0.030*	
C7	0.4062 (3)	0.5249 (2)	0.69742 (17)	0.0219 (6)	
H7	0.4326	0.4781	0.6562	0.026*	
C8	0.2941 (3)	0.5922 (2)	0.68141 (17)	0.0232 (6)	
H8	0.2633	0.6365	0.7224	0.028*	
C9	0.2165 (3)	0.6014 (2)	0.60506 (17)	0.0218 (6)	
C10	0.2406 (3)	0.5114 (2)	0.54359 (16)	0.0196 (6)	
C11	0.1878 (2)	0.3965 (2)	0.54427 (16)	0.0196 (6)	
C12	0.2102 (2)	0.3183 (2)	0.48211 (16)	0.0192 (6)	
C13	0.1502 (3)	0.2022 (2)	0.47167 (16)	0.0211 (6)	
H13	0.0911	0.1735	0.5063	0.025*	
C14	0.1783 (3)	0.1328 (2)	0.41150 (17)	0.0237 (6)	
C15	0.2654 (3)	0.1713 (2)	0.35909 (17)	0.0255 (6)	
H15	0.2848	0.1199	0.3184	0.031*	
C16	0.3223 (3)	0.2835 (2)	0.36714 (17)	0.0242 (6)	
H16	0.3817	0.3100	0.3320	0.029*	
C17	0.2931 (3)	0.3600 (2)	0.42726 (16)	0.0206 (6)	
C18	0.3167 (3)	0.5479 (2)	0.48365 (17)	0.0215 (6)	
C19	0.3681 (3)	0.6752 (2)	0.48052 (18)	0.0269 (6)	
H19A	0.4366	0.6794	0.4448	0.040*	
H19B	0.4088	0.7001	0.5321	0.040*	
H19C	0.2923	0.7290	0.4626	0.040*	
C20	0.1112 (3)	0.3561 (2)	0.60680 (16)	0.0210 (6)	
C21	−0.0006 (3)	0.4208 (2)	0.62611 (17)	0.0238 (6)	
H21	−0.0310	0.4900	0.5969	0.029*	
C22	−0.0675 (3)	0.3855 (3)	0.68711 (18)	0.0288 (7)	
H22	−0.1427	0.4308	0.6999	0.035*	
C23	−0.0251 (3)	0.2841 (3)	0.72961 (18)	0.0306 (7)	
H23	−0.0697	0.2607	0.7723	0.037*	
C24	0.0828 (3)	0.2169 (2)	0.70960 (18)	0.0293 (7)	
H24	0.1099	0.1458	0.7377	0.035*	
C25	0.1511 (3)	0.2522 (2)	0.64943 (17)	0.0250 (6)	
H25	0.2256	0.2059	0.6367	0.030*	

### Atomic displacement parameters (Å^2^)

**Table:**

	*U*^11^	*U*^22^	*U*^33^	*U*^12^	*U*^13^	*U*^23^
Cl1	0.0373 (4)	0.0150 (3)	0.0400 (5)	−0.0062 (3)	0.0013 (3)	−0.0048 (3)
O1	0.0212 (10)	0.0136 (9)	0.0452 (13)	0.0019 (7)	0.0001 (9)	−0.0028 (8)
N1	0.0180 (11)	0.0158 (11)	0.0339 (14)	0.0000 (9)	0.0036 (9)	0.0010 (9)
C1	0.0213 (13)	0.0120 (12)	0.0325 (16)	−0.0028 (10)	0.0048 (11)	0.0020 (10)
C2	0.0247 (14)	0.0163 (13)	0.0354 (17)	0.0002 (10)	0.0042 (12)	−0.0017 (11)
C3	0.0313 (16)	0.0166 (13)	0.0342 (17)	−0.0025 (11)	0.0026 (12)	−0.0023 (11)
C4	0.0320 (16)	0.0165 (13)	0.0348 (17)	−0.0024 (11)	−0.0034 (13)	0.0032 (11)
C5	0.0248 (15)	0.0170 (13)	0.0442 (19)	0.0037 (11)	−0.0011 (13)	0.0019 (12)
C6	0.0255 (14)	0.0172 (13)	0.0340 (17)	0.0015 (11)	0.0060 (12)	−0.0009 (11)
C7	0.0221 (14)	0.0118 (11)	0.0326 (16)	−0.0023 (10)	0.0070 (11)	−0.0002 (11)
C8	0.0245 (14)	0.0155 (12)	0.0302 (16)	0.0010 (10)	0.0056 (11)	−0.0036 (11)
C9	0.0162 (13)	0.0122 (12)	0.0371 (16)	−0.0043 (10)	0.0038 (11)	−0.0005 (11)
C10	0.0153 (12)	0.0151 (12)	0.0279 (15)	0.0016 (10)	0.0004 (10)	0.0016 (10)
C11	0.0126 (12)	0.0154 (12)	0.0298 (15)	0.0007 (9)	−0.0014 (10)	0.0019 (10)
C12	0.0115 (12)	0.0158 (12)	0.0298 (15)	0.0020 (9)	0.0005 (10)	0.0012 (10)
C13	0.0156 (12)	0.0153 (12)	0.0320 (16)	0.0011 (10)	0.0018 (11)	0.0017 (11)
C14	0.0237 (14)	0.0134 (12)	0.0326 (16)	0.0009 (10)	−0.0023 (11)	0.0009 (11)
C15	0.0283 (15)	0.0171 (13)	0.0310 (16)	0.0054 (11)	0.0035 (12)	−0.0025 (11)
C16	0.0196 (13)	0.0209 (13)	0.0324 (16)	0.0011 (11)	0.0039 (11)	0.0009 (11)
C17	0.0139 (12)	0.0148 (12)	0.0329 (16)	0.0013 (9)	0.0017 (10)	0.0014 (11)
C18	0.0163 (13)	0.0143 (12)	0.0332 (16)	−0.0002 (10)	0.0002 (11)	0.0014 (11)
C19	0.0246 (14)	0.0149 (13)	0.0416 (18)	−0.0036 (11)	0.0059 (12)	0.0009 (12)
C20	0.0191 (13)	0.0138 (12)	0.0294 (15)	−0.0042 (9)	0.0003 (11)	−0.0011 (10)
C21	0.0182 (13)	0.0177 (13)	0.0348 (17)	−0.0018 (10)	0.0013 (11)	−0.0017 (11)
C22	0.0206 (14)	0.0254 (14)	0.0409 (18)	−0.0062 (11)	0.0066 (12)	−0.0082 (13)
C23	0.0322 (16)	0.0280 (15)	0.0329 (17)	−0.0155 (13)	0.0085 (13)	−0.0038 (12)
C24	0.0374 (17)	0.0172 (13)	0.0319 (17)	−0.0087 (12)	−0.0007 (13)	0.0029 (12)
C25	0.0273 (14)	0.0134 (12)	0.0334 (17)	−0.0018 (10)	0.0000 (12)	−0.0012 (11)

### Geometric parameters (Å, °)

**Table:**

Cl1—C14	1.739 (3)	C12—C17	1.417 (4)
O1—C9	1.228 (3)	C12—C13	1.422 (3)
N1—C18	1.314 (4)	C13—C14	1.360 (4)
N1—C17	1.379 (3)	C13—H13	0.9500
C1—C2	1.396 (4)	C14—C15	1.403 (4)
C1—C6	1.399 (4)	C15—C16	1.368 (4)
C1—C7	1.457 (4)	C15—H15	0.9500
C2—C3	1.379 (4)	C16—C17	1.409 (4)
C2—H2	0.9500	C16—H16	0.9500
C3—C4	1.391 (4)	C18—C19	1.506 (3)
C3—H3	0.9500	C19—H19A	0.9800
C4—C5	1.382 (4)	C19—H19B	0.9800
C4—H4	0.9500	C19—H19C	0.9800
C5—C6	1.384 (4)	C20—C21	1.399 (4)
C5—H5	0.9500	C20—C25	1.402 (4)
C6—H6	0.9500	C21—C22	1.381 (4)
C7—C8	1.340 (4)	C21—H21	0.9500
C7—H7	0.9500	C22—C23	1.384 (4)
C8—C9	1.457 (4)	C22—H22	0.9500
C8—H8	0.9500	C23—C24	1.385 (4)
C9—C10	1.508 (4)	C23—H23	0.9500
C10—C11	1.380 (3)	C24—C25	1.377 (4)
C10—C18	1.425 (4)	C24—H24	0.9500
C11—C12	1.429 (4)	C25—H25	0.9500
C11—C20	1.477 (4)		
			
C18—N1—C17	117.8 (2)	C13—C14—C15	122.3 (2)
C2—C1—C6	118.3 (3)	C13—C14—Cl1	119.8 (2)
C2—C1—C7	123.4 (2)	C15—C14—Cl1	117.8 (2)
C6—C1—C7	118.3 (3)	C16—C15—C14	119.4 (3)
C3—C2—C1	120.9 (3)	C16—C15—H15	120.3
C3—C2—H2	119.6	C14—C15—H15	120.3
C1—C2—H2	119.6	C15—C16—C17	120.3 (3)
C2—C3—C4	120.1 (3)	C15—C16—H16	119.8
C2—C3—H3	119.9	C17—C16—H16	119.8
C4—C3—H3	119.9	N1—C17—C16	117.3 (2)
C5—C4—C3	119.9 (3)	N1—C17—C12	122.7 (2)
C5—C4—H4	120.1	C16—C17—C12	119.9 (2)
C3—C4—H4	120.1	N1—C18—C10	123.1 (2)
C4—C5—C6	120.0 (3)	N1—C18—C19	116.4 (3)
C4—C5—H5	120.0	C10—C18—C19	120.5 (2)
C6—C5—H5	120.0	C18—C19—H19A	109.5
C5—C6—C1	120.8 (3)	C18—C19—H19B	109.5
C5—C6—H6	119.6	H19A—C19—H19B	109.5
C1—C6—H6	119.6	C18—C19—H19C	109.5
C8—C7—C1	126.9 (3)	H19A—C19—H19C	109.5
C8—C7—H7	116.6	H19B—C19—H19C	109.5
C1—C7—H7	116.6	C21—C20—C25	118.3 (3)
C7—C8—C9	124.0 (3)	C21—C20—C11	121.4 (2)
C7—C8—H8	118.0	C25—C20—C11	120.4 (2)
C9—C8—H8	118.0	C22—C21—C20	120.9 (3)
O1—C9—C8	121.3 (2)	C22—C21—H21	119.6
O1—C9—C10	119.3 (3)	C20—C21—H21	119.6
C8—C9—C10	119.4 (2)	C21—C22—C23	120.1 (3)
C11—C10—C18	120.5 (2)	C21—C22—H22	120.0
C11—C10—C9	120.8 (2)	C23—C22—H22	120.0
C18—C10—C9	118.7 (2)	C22—C23—C24	119.7 (3)
C10—C11—C12	117.3 (2)	C22—C23—H23	120.2
C10—C11—C20	121.2 (2)	C24—C23—H23	120.2
C12—C11—C20	121.5 (2)	C25—C24—C23	120.7 (3)
C17—C12—C13	118.6 (2)	C25—C24—H24	119.7
C17—C12—C11	118.3 (2)	C23—C24—H24	119.7
C13—C12—C11	123.0 (2)	C24—C25—C20	120.4 (3)
C14—C13—C12	119.3 (3)	C24—C25—H25	119.8
C14—C13—H13	120.4	C20—C25—H25	119.8
C12—C13—H13	120.4		
			
C6—C1—C2—C3	−1.5 (4)	C13—C14—C15—C16	−1.5 (4)
C7—C1—C2—C3	176.5 (3)	Cl1—C14—C15—C16	176.6 (2)
C1—C2—C3—C4	0.8 (4)	C14—C15—C16—C17	−0.3 (4)
C2—C3—C4—C5	0.2 (4)	C18—N1—C17—C16	178.5 (2)
C3—C4—C5—C6	−0.6 (4)	C18—N1—C17—C12	−0.3 (4)
C4—C5—C6—C1	−0.1 (4)	C15—C16—C17—N1	−175.5 (2)
C2—C1—C6—C5	1.1 (4)	C15—C16—C17—C12	3.3 (4)
C7—C1—C6—C5	−177.0 (3)	C13—C12—C17—N1	174.2 (2)
C2—C1—C7—C8	0.2 (4)	C11—C12—C17—N1	−4.0 (4)
C6—C1—C7—C8	178.2 (3)	C13—C12—C17—C16	−4.5 (4)
C1—C7—C8—C9	176.9 (2)	C11—C12—C17—C16	177.2 (2)
C7—C8—C9—O1	−163.7 (3)	C17—N1—C18—C10	4.0 (4)
C7—C8—C9—C10	14.7 (4)	C17—N1—C18—C19	−176.0 (2)
O1—C9—C10—C11	−104.5 (3)	C11—C10—C18—N1	−3.2 (4)
C8—C9—C10—C11	77.1 (3)	C9—C10—C18—N1	178.2 (2)
O1—C9—C10—C18	74.1 (3)	C11—C10—C18—C19	176.8 (2)
C8—C9—C10—C18	−104.3 (3)	C9—C10—C18—C19	−1.9 (4)
C18—C10—C11—C12	−1.3 (4)	C10—C11—C20—C21	54.1 (4)
C9—C10—C11—C12	177.3 (2)	C12—C11—C20—C21	−126.0 (3)
C18—C10—C11—C20	178.7 (2)	C10—C11—C20—C25	−124.8 (3)
C9—C10—C11—C20	−2.7 (4)	C12—C11—C20—C25	55.2 (3)
C10—C11—C12—C17	4.6 (3)	C25—C20—C21—C22	2.0 (4)
C20—C11—C12—C17	−175.4 (2)	C11—C20—C21—C22	−176.9 (2)
C10—C11—C12—C13	−173.6 (2)	C20—C21—C22—C23	−0.7 (4)
C20—C11—C12—C13	6.5 (4)	C21—C22—C23—C24	−1.4 (4)
C17—C12—C13—C14	2.7 (4)	C22—C23—C24—C25	2.1 (4)
C11—C12—C13—C14	−179.1 (2)	C23—C24—C25—C20	−0.8 (4)
C12—C13—C14—C15	0.3 (4)	C21—C20—C25—C24	−1.2 (4)
C12—C13—C14—Cl1	−177.80 (19)	C11—C20—C25—C24	177.6 (2)

### Hydrogen-bond geometry (Å, °)

**Table:**

Cg1 is the centroid of the N1,C10–C12,C17,C18 ring.

**Table:**

*D*—H···*A*	*D*—H	H···*A*	*D*···*A*	*D*—H···*A*
C5—H5···O1^i^	0.95	2.48	3.315 (3)	146
C21—H21···Cg1^ii^	0.95	2.71	3.459 (3)	137

Symmetry codes: (i) −*x*+1, *y*−1/2, −*z*+3/2; (ii) −*x*, −*y*+1, −*z*+1.
